# Evolutionary changes in gene expression profiles associated with the coevolution of male and female genital parts among closely related ground beetle species

**DOI:** 10.1186/s12864-022-08865-2

**Published:** 2022-09-08

**Authors:** Shota Nomura, Teiji Sota

**Affiliations:** 1 Department of Zoology, Graduate School of Science, Kyoto University, Kyoto, Sakyo 606-8502 Japan; 2 Division of Evolutionary Developmental Biology, National Institute for Basic Biology, 38, Nishigonaka, Okazaki, Myodaiji 444- 8585 Japan

**Keywords:** Character evolution, Differentially expressed genes, Genital formation, Interspecific differences, Sexual traits, Transcriptome

## Abstract

**Background:**

The coevolutionary dynamics of corresponding male and female sexual traits, including genitalia, may be driven by complex genetic mechanisms. *Carabus* (*Ohomopterus*) ground beetles show correlated evolution in the size of their functionally corresponding male and female genital parts. To reveal the genetic mechanisms involved in the evolution of size, we investigated interspecific differences in gene expression profiles in four closely related species (two species each with long and short genital parts) using transcriptome data from genital tissues in the early and late pupal stages.

**Results:**

We detected 1536 and 1306 differentially expressed genes (DEGs) among the species in males and 546 and 1959 DEGs in females in the two pupal stages, respectively. The DEGs were clustered by species-specific expression profiles for each stage and sex to identify candidate gene clusters for genital size based on the expression patterns among the species and gene ontology. We identified one and two gene clusters in females and males, respectively, all from the late pupal stage; one cluster of each sex showed similar expression profiles in species with similar genital size, which implies a common gene expression change associated with similar genital size in each sex. However, the remaining male cluster showed different expression profiles between species with long genital parts, which implies species-specific gene expression changes. These clusters did not show sex-concordant expression profiles for genital size differences.

**Conclusion:**

Our study demonstrates that sex-independent and partly species-specific gene expression underlies the correlated evolution of male and female genital size. These results may reflect the complex evolutionary history of male and female genitalia.

**Supplementary Information:**

The online version contains supplementary material available at 10.1186/s12864-022-08865-2.

## Background

The evolution and diversification of sexual traits related to mating and fertilization success may drive reproductive isolation among species and has been a significant subject in evolutionary studies [1–4]. An important question related to this subject is how evolutionary changes have occurred in diversified male and female sexual traits during the phylogenetic divergence of related species. Because both sexual and natural selection are involved in the coevolutionary dynamics of male and female traits, their evolutionary paths are likely to be complex [1, 2, 5]. The evolutionary changes in sexual traits that have occurred with speciation can be clarified by studying the genetic mechanisms underlying species-specific male and female sexual traits among closely related species. However, few comparative studies have been conducted on the genetic control of sexual traits among closely related species.

Male and female genitalia of animals with internal fertilization are notable sexual traits that show marked diversification and coevolution between the sexes among closely related species in some animal lineages [6, 7]. While male and female genitalia require morphological matching to ensure mating and fertilization success, various factors such as natural selection to avoid hybridization or predation, female choice, and sexual conflict can drive the coevolution of male and female genital morphology [7, 8]. The differences in genital size and shape among populations resulting from genital coevolution between the sexes may reinforce reproductive isolation and promote speciation [9–12]. Because selective factors and their strengths driving genital coevolution vary among populations, related populations can evolve independently into different morphological states of male and female genitalia. The evolutionary trends of male and female genital morphology, such as size, may not reflect the species phylogeny. Thus, species-specific genital sizes and shapes may not show gradual changes and may show independent or parallel evolution even in closely related species. Regarding the genetic background of genital evolution, similar genital sizes and shapes among closely related species may have formed based on identical or different genetic variation and changes in expression. However, the genetic background of genital coevolution between the sexes remains largely unknown.

In Japan, ground beetles of the subgenus *Ohomopterus* (genus *Carabus*) exhibit coevolution between corresponding male and female genital parts, copulatory pieces, and vaginal appendices among 17 species [13–15]. During copulation, the copulatory pieces of the corresponding size and shape are inserted into the vaginal appendices to ensure successful copulation and fertilization [16, 17]. The species-specific size and shape of these genital parts contribute to mechanical reproductive isolation among species, although it is not strong enough to avoid interspecific copulation completely [11, 18, 19]. In *Ohomopterus*, the ancestral form of the male copulatory piece is inferred to have been a small triangle, from which a wider pentagonal type evolved, and elongation and shortening of copulatory pieces occurred repeatedly in the derived species group, named the *iwawakianus*-*insulicola* group [20]. The evolution of the shape and size of female genital parts occurred in parallel with male genital parts. The *iwawakianus*-*insulicola* group comprises three and four species with short and elongated genital parts, respectively. This species group has been the subject of evolutionary studies for genital evolution in terms of selection factors and genetic backgrounds. Previous studies in this species group have suggested that selection for morphological matching between the sexes, sexual conflict associated with male genital evolution under sperm competition, and selection to avoid hybridization have affected the coevolution of the size and shape of male and female genital parts [11, 17, 21, 22]. The genetic background underlying the evolution of species-specific genital size and shapes has been investigated mainly in *Carabus maiyasanus*, *C*. *iwawakianus*, and *C*. *uenoi*, which occur in the western part of the range of the *iwawakianus*-*insulicola* group [14, 15, 23, 24]. Fujisawa et al. [15] performed a genomic comparison with QTL mapping analyses and detected six genomic regions and 21 genes that might control male and female genital size in *C*. *iwawakianus* and *C*. *maiyasanus*. Nomura et al. [24] compared gene expression profiles in genital morphogenesis among the three species and suggested that different sets of genes showing sex-discordant expression changes were important for the evolution of genital size in *C*. *maiyasanus* and *C*. *iwawakianus*, which had long and short genital parts, respectively. By contrast, sex-concordant expression changes in some genes were important in the coevolution of extremely long male and female genitalia in *C*. *uenoi*. These findings imply that genetic mechanisms underlying the coevolution of genital size between the sexes differ even among closely related species. However, because all three species in previous studies had different genital size, it is unclear whether the same or different genetic changes are essential for forming similar genital size among closely related species.

**Fig. 1:**
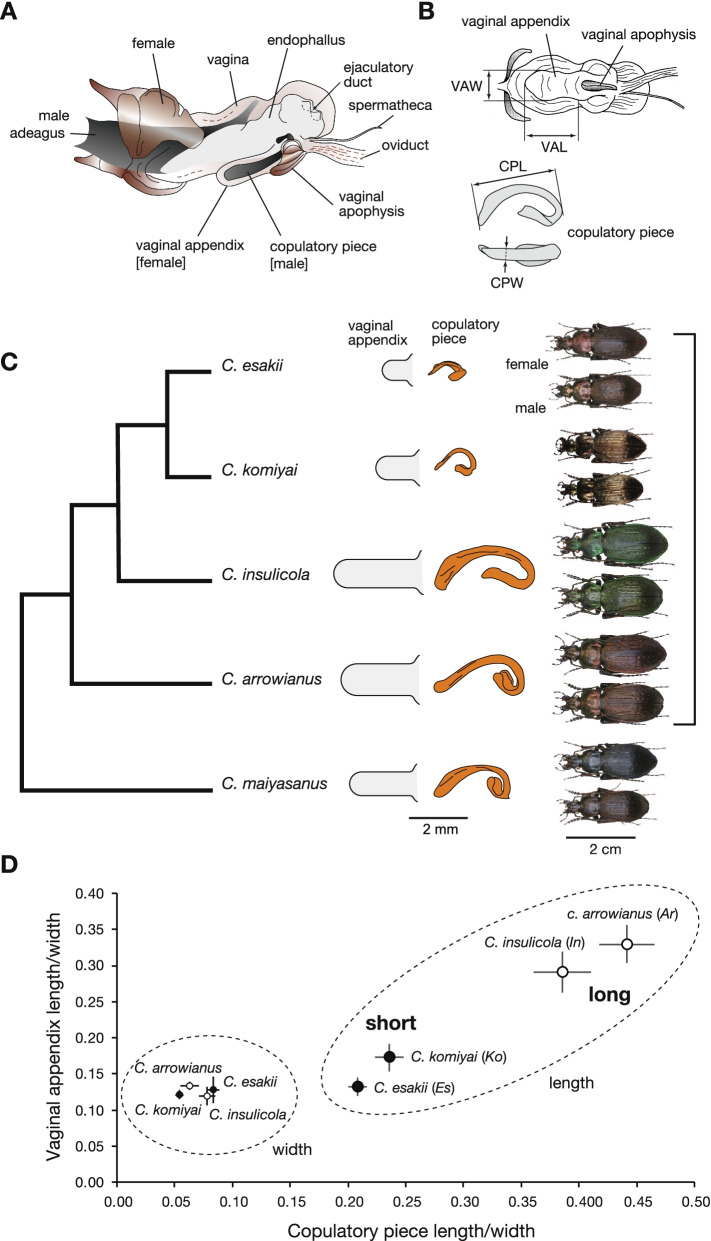
Male and female genitalia and phylogenetic relationships of *Ohomopterus* species used in this study. **A** Genital system and coupling of female and male genitalia in *Ohomopterus* beetles. **B** Measurements of the length and width of the vaginal appendix (upper) and copulatory piece (lower). **C** Phylogenetic relationships inferred from a restriction site associated DNA sequence data [15], vaginal appendix, copulatory piece, and habitus of adult beetles (upper: female; lower: male) of the four study species and the outgroup species, *Carabus maiyasanus*. **D** Relationships between length/width of the copulatory piece and vaginal appendix in the four study species. Photographs and illustrations by T. Sota

In the eastern part of the range of the *iwawakianus*-*insulicola* group, there are four parapatric species: *Carabus arrowianus*, *C*. *insulicola*, *C*. *komiyai*, and *C*. *esakii*. *C*. *komiyai* and *C*. *esakii* have short genital parts whereas *C*. *arrowianus* and *C*. *insulicola* have long ones (Fig. 1). Fujisawa et al. [15] examined genomic sequence divergence between the short- and long-genital species (*C*. *komiyai* vs. *C*. *arrowianus*, and *C*. *esakii* vs. *C*. *insulicola*) for the genital QTL regions of the *C*. *iwawakianus*–*C*. *maiyasanus* pair and found that the QTL regions of *C*. *iwawakianus*–*C*. *maiyasanus* have similarly diverged in the above two species pairs. This finding implies that some common genomic regions are responsible for the parallel evolution of genital size [15]. Divergence of gene expression profiles in sets of genes involved in genital morphogenesis may also occur and affect genital size, and the genes and expression profiles may be shared among species with similar genital size. By comparing gene expression profiles during genital morphogenesis among the four species, we may be able to clarify common genetic changes in the gene expression involved in the formation of long and short genital parts in males and females.

In this study, we investigated the gene expression changes associated with the interspecific differences in coevolving male and female genital size (lengths) based on comparisons of gene expression profiles in *C*. *arrowianus* (Ar), *C*. *insulicola* (In), *C*. *komiyai* (Ko), and *C*. *esakii* (Es) (Fig. 1). We assumed that interspecific differences in genital size are based on different gene expression profiles during genital morphogenesis among species. We obtained transcriptomes from the pupal stage, during which the formation of genital parts occurs [25], and analyzed differentially expressed genes (DEGs) among the species for each sex because male and female genitalia are not strictly homologous and the genes involved in genital part length may show sex-specific expression profiles. For DEGs among species in each sex, we used a clustering method and divided the DEGs into groups sharing similar expression profiles. In the four species used in this study, the evolution of genital size (length) appears to have occurred only once in the common ancestor of *C*. *esakii* and *C*. *komiyai*, from long to short parts (Fig. 1). Therefore, we focused on the clusters with differences in the expression levels between species with long (Ar and In) and short (Ko and Es) genital parts. At the same time, because clusters of DEGs (hereafter, gene clusters) containing genes that might be important for genital formation may also be involved in differences in genital length, we performed gene ontology (GO) enrichment analyses on all gene clusters to clarify which contained DEGs that play important roles in genital formation. Finally, we identified candidate genes for the differences in genital lengths in the gene clusters selected based on the above two criteria among transcription factors (TFs) and genes involved in “imaginal disc development” and “cuticle development”. We found candidate genes for the differences in vaginal appendix length, which showed similar expression profiles among species with similar vaginal appendix sizes in females. For copulatory piece of males, however, we found two groups of candidate genes, which showed either similar or different expression profiles among species with similar copulatory piece lengths. Our results imply that the evolution of genital length may not necessarily have occurred through the same (or shared) gene expression changes in each sex.

## Results

### Draft genome assembly

We obtained 720 million Chromium linked reads of a male *C*. *esakii* genome (Table S1) and performed draft genome assembly with 746,666 linked reads (56 × coverage of the expected genome size, 200 Mbp; see Methods) using the Supernova2 assembler. The assembled genome was 193 Mbp in length and 2.0 Mbp in contig N50 (Table 1); it showed high completeness (98.2%) in terms of BUSCO score, and 23,767 protein-coding genes were predicted by using the Braker2 pipeline (Table 1). We used this draft genome as the reference genome in the following analyses.

**Table 1:** Assembly statistics of the *Carabus* (*Ohomopterus*) *esakii* genome

Number of contigs	12,194
Number of contigs > 10kbp	932
Total length of contigs (bp)	193,159,710
Total length of contigs > 10kbp (bp)	159,230,195
Average contig length (bp)	15,841
Average contig length > 10kbp (bp)	170,848
Longest contig length (bp)	7,158,272
Shortest contig length (bp)	1,000
N50 contig length (bp)	2,023,420
GC contents (%)	34.66
BUSCO score: complete (%)	98.2 (single: 96.8, duplicated: 1.4)
BUSCO score: fragmented (%)	0.8
BUSCO score: missing (%)	1.0
Number of protein coding genes	23,767

### Transcriptomic data analyses of all genes

We obtained transcriptomic data by RNA-seq for male and female pupae of *C*. *arrowianus* (Ar), *C*. *insulicola* (In), *C*. *komiyai* (Ko), and *C*. *esakii* (Es) from genital tissue of two pupal stages: the early pupal stage (PE) at 1–3 days after pupation and the late pupal stage (PL) at 4–6 days after pupation (*n* = 3 for each sex of each species; total number of transcriptome samples, 48; Fig. 1; Table S2). These pupal stages presumably corresponded to before and during formation of the copulatory piece and vaginal appendix [25]. We mapped all of the transcriptome sequence reads to the *C*. *esakii* draft genome. There was no mapping bias to *C. esakii* genome among the species (see Methods). We found that 9,778 genes were mapped with > 10 reads per sample among 48 samples and used these genes in the following analyses. Expression variation analyses of these genes showed mean percentages of expression variance that were explained by species, stage, and sex of 10.1% (max, 77.0%), 8.86% (max, 64.3%), and 3.03% (max, 71.9%), respectively (Fig. S1). Principal components analyses (PCA) of the expression profiles of these genes showed that individual samples were not clustered discretely by species, stage, and sex (Fig. S2). However, there were significant differences in principal component scores 1 (PC1) and 2 (PC2) among species, between stages, and between sexes (Table S3). Thus, the effects of species, stage, and sex on the variation in gene expression were limited.

### DEGs among species and gene clusters of DEGs

**Fig. 2:**
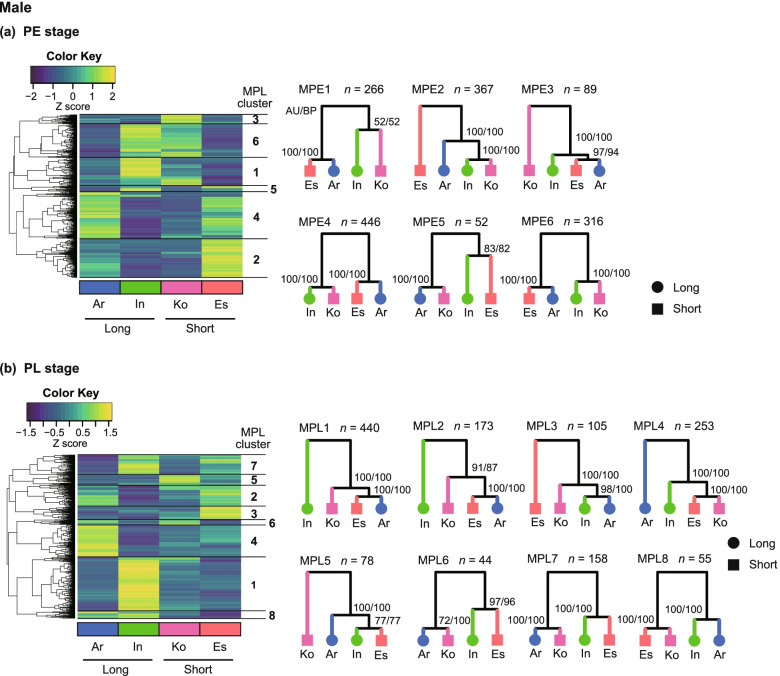
Hierarchical clustering and expression patterns in each cluster in males. **A** Early pupal (PE) stage; **B** Late pupal (PL) stage. Heatmaps are based on the expression profiles of interspecific DEGs, and the clusters are based on the similarity of expression profiles among the species. The yellow to blue colour gradient of the heatmap corresponds to the gradient of higher to lower expression levels of genes. The tree diagrams on the right show the species clustering based on the similarity of expression profiles of DEGs in each cluster. The *n* value indicates the number of DEGs included in each cluster. The numerals shown on each node are approximately unbiased *P*-values followed by the bootstrap percentage (AU/BP). Species: Ar, *C*. *arrowianus*; In, *C*. *insulicola*; Ko, *C*. *komiyai*; and Es, *C*. *esakii*. Ar and In are species with long copulatory pieces, and Ko and Es have short copulatory pieces

**Fig. 3:**
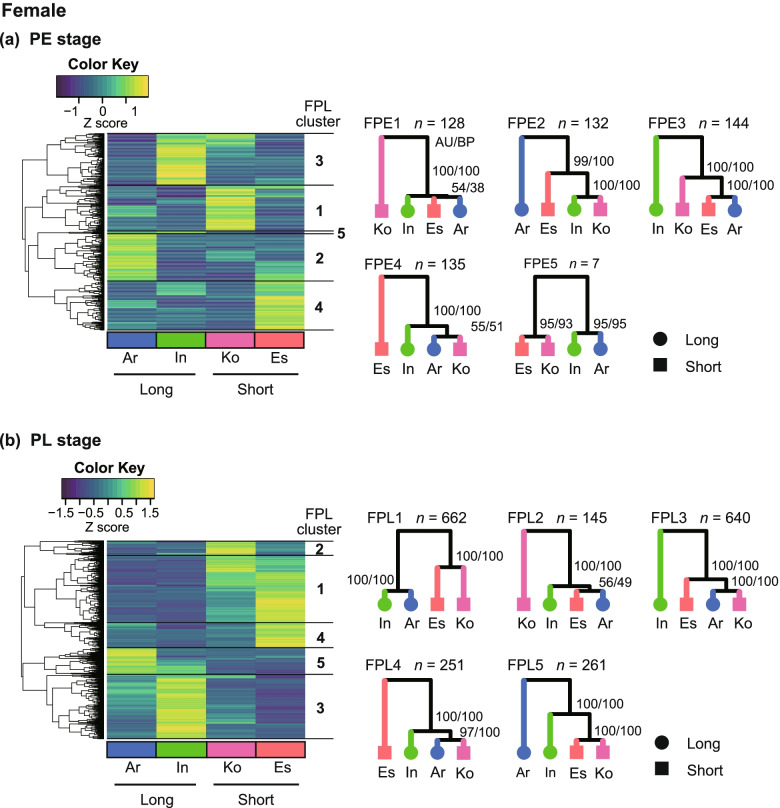
Hierarchical clustering and expression patterns in each cluster in females. **A** Early pupal (PE) stage; **B** Late pupal (PL) stage. Heatmaps are based on the expression profiles of interspecific DEGs, and the clusters are based on the similarity of the expression profiles among the species. The yellow to blue colour gradient of the heatmap corresponds to the gradient of higher to lower expression levels of the genes. The tree diagrams on the right show the species clustering based on the similarity of expression profiles of DEGs in each cluster. The *n* value indicates the number of DEGs included in each cluster. The numerals shown on each node are approximately unbiased *P*-values followed by the bootstrap percentage (AU/BP). Species: Ar, *C*. *arrowianus*; In, *C*. *insulicola*; Ko, *C*. *komiyai*; and Es, *C*. *esakii*. Ar and In are species with long vaginal appendices, and Ko and Es have short copulatory pieces

To investigate differences in gene expression between species associated with length differences in the copulatory piece and vaginal appendix, we compared gene expressions for each stage and sex among the four species. We found 1536 and 1306 DEGs (false discovery rate [FDR] < 0.05) in the PE and PL stages of males, respectively, and 546 and 1959 DEGs in the PE and PL stages of females, respectively. We classified these DEGs according to the different expression profiles among the species for each stage and sex using a hierarchical clustering method and constructed dendrograms that showed the similarity of the expression profiles among the four species (Figs. 2 and 3). Clusters of DEGs that were separated by distances (height) > 3 from one another were arbitrarily defined as clusters for candidate gene searches (Figs. S3, S4).

In the male PE stage (MPE), the DEGs were divided into six clusters; in all of these clusters, pairs of species with a long and a short copulatory piece showed similar expression patterns, and no cluster contained pairs of species with similar copulatory piece length that showed similar expression profiles (Fig. 2a). In the male PL stage (MPL), the DEGs were divided into eight clusters (MPL1–MPL8), and the DEGs in MPL8 showed a divergence between the species with long (Ar and In) and short (Ko and Es) copulatory pieces (Fig. 2b). The expression profiles of DEGs in the MPL3 cluster were similar between Ar and In, and the DEGs in the MPL4 cluster were similar between Ko and Es.

In the female PE stage (FPE), the DEGs were divided into five clusters, and the DEGs in the FPE5 cluster showed similar expression profiles between Ar and In and between Ko and Es, which were species pairs with similar vaginal appendix lengths. However, the FPE5 cluster contained only seven genes (Fig. 3a). The expression profiles of the DEGs in the other clusters did not show similar expression between Ar and In or between Ko and Es. In the female PL stage (FPL), DEGs were divided into five clusters, and the DEGs in the FPL1 cluster showed similar expression between Ar and In, and between Ko and Es, and the DEGs in the FPL5 cluster showed similar expression profiles between Ko and Es (Fig. 3b).

We assume that the DEGs in the clusters with similar expression profiles between two species with similar genital lengths, Ar and In (long) and Ko and Es (short), are potentially involved in the development of genital parts with different lengths among species. The MPL8 and FPL1 clusters showed this long vs. short genital pattern, and these were included in the search for genes involved in the genital differences.

### Gene clusters potentially related to genital morphogenesis and interspecific genital size differences

We performed GO enrichment analyses to identify clusters of DEGs enriched with functions related to genital development and thus potentially related to interspecific genital size differences. We assumed that genes involved in “imaginal disc development” and “cuticle development” were involved in genital development. The “imaginal disc development” includes genital, leg and wing disc development processes, in which some common genes are used in *Drosophila* [26]. “Cuticle development” was another candidate term since genes involved in cuticle development and projection were related to the species-specific genital morphology in other *Ohomopterus* species [15]. In the male clusters, MPE2, MPE4, and MPL4 were enriched with GO terms for imaginal disc development, and the MPL4 cluster was also enriched with GO terms for cuticle development (Table 2). The FPL1 cluster was enriched with GO terms for imaginal disc development and cuticle development in the female clusters. Among the genes for “imaginal disc development,” genes for "genital disc development" were few and not enriched in any clusters (Table 2). The primary genes for genital disc development in insects, *doublesex* (*dsx*) and *Abdominal-B* (*Abd-B*) were found in the male cluster MPE2 (*Abd-B*) and in the female clusters FPE2 (*dsx*) and FPL1 (*Abd-B*).

**Table 2:** Number of genes included in the GO terms putatively related to genital morphology in each cluster. Bold numbers indicate that the terms are significantly enriched in the clusters (FDR < 0.01). Note that the GO term “genital disc development” is included in “imaginal disc development”

	PE Male cluster							
	MPE1	MPE2	MPE3	MPE4	MPE5	MPE6		
all	266	367	89	446	52	316		
transcription factors	7	19	2	30	1	11		
*GO term*								
imaginal disc development	10	**47**	4	**47**	0	3		
[genital disc development]	1	5	0	4	0	0		
cuticle development	4	4	1	9	0	2		
	PL Male cluster							
	MPL1	MPL2	MPL3	MPL4	MPL5	MPL6	MPL7	MPL8
all	440	173	105	253	78	44	158	55
transcription factors	15	10	3	5	0	1	4	1
*GO term*								
imaginal disc development	6	9	4	**29**	1	2	4	0
[genital disc development]	0	1	1	1	0	0	0	0
cuticle development	4	5	3	**13**	0	0	1	1
	PE Female cluster							
	FPE1	FPE2	FPE3	FPE4	FPE5			
all	128	132	144	135	7			
transcription factors	2	6	1	2	0			
*GO term*								
imaginal disc development	0	8	2	4	0			
[genital disc development]	0	3	0	1	0			
cuticle development	0	8	2	2	0			
	PL Female cluster							
	FPL1	FPL2	FPL3	FPL4	FPL5			
all	662	145	640	251	261			
transcription factors	15	2	18	8	7			
*GO term*								
imaginal disc development	**48**	2	12	21	9			
[genital disc development]	1	0	0	3	0			
cuticle development	**21**	6	2	10	3			

Thus, we selected MPE2, MPE4, MPL4, FPE2, and FPL1 as the candidate gene clusters for genital morphogenesis. Of these, MPE2, MPE4, and FPE2 did not show similar gene expression profiles between species with similar genital lengths, and hence these might not be related to the genital differences. Therefore, MPE2, MPE4, and FPE2 were excluded from the following analyses. The remaining MPL4 and FPL1 male and female candidate gene clusters shared 105 genes, which accounted for 41.5% of genes in MPL4.

### DEGs involved in interspecific differences in genital size

Having identified two clusters in males (MPL4, MPL8) and two clusters in females (FPE5, FPL1) as candidate clusters that may contain genes involved in differences in genital part length, we focused on genes with the GO terms “imaginal disc development” and “cuticle development”. We also focused on TFs because these are directly involved in regulating the expression of many genes and are important for organ development. Expression changes in TFs can be primary factors involved in differences in genital size. We specifically focused on whether significant differences existed between species with long vs. short genital parts in our comparison of expression patterns. When the expression profiles were not explained by differences in genital length, we investigated which of the four comparisons between species with different genital lengths (Ar vs. Ko, Ar vs. Es, In vs. Ko, and In vs. Es) showed significantly different expression profiles.

**Fig. 4:**
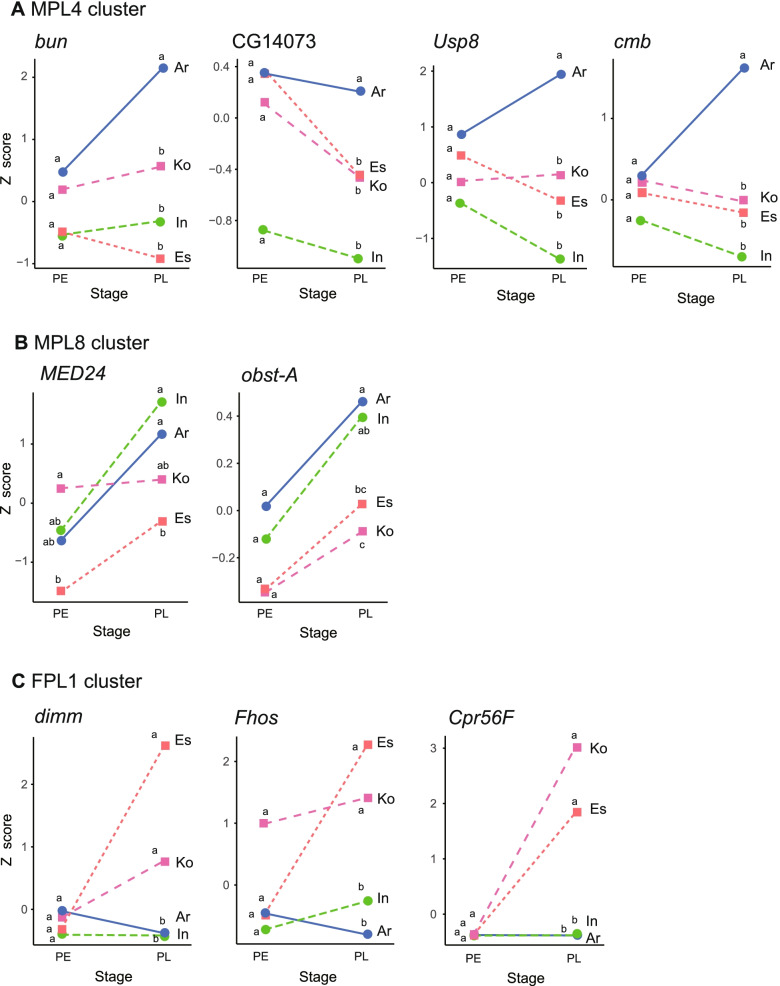
Expression profiles of the candidate genes included in candidate clusters. The candidate genes for interspecific differences in copulatory piece length (**A**, **B**) and vaginal appendix length (**C**). **A** MPL4 cluster genes, *bun*, *CG14073*, *Usp8*, and *cmb*. **B** MPL8 cluster genes, *MED24*, and *obst-A*. **C** FPL1 cluster genes, *dimm*, *Sb*, *Pka-C3*, *Fhos*, and *Cpr56F*. Species: Ar, *C*. *arrowianus*; In, *C*. *insulicola*; Ko, *C*. *komiyai*; and Es, *C*. *esakii*. Expression levels with the same letter (a, b, c) are not significantly different from each other (*P* > 0.05) among species in each stage by the multiple comparison test

In males, MPL4 contained 5 TFs, 29 genes with the GO term “imaginal disc development”, and 13 genes with the GO term “cuticle development” (Table S4). Of these, the expression levels of a TF (*bunched* [*bun*]) and three genes with imaginal disc development (*CG14073*, *Ubiquitin specific protease 8* [*Usp8*], and *combover* [*cmb*]) were significantly different in the comparisons between one of the species with long genital parts (Ar) and those with short genital parts (Ko and Es) in the PL stage. No genes showed significant differences in the other species with long genital parts (In) and those with short genital parts. These four genes in MPL4 showed higher expression levels in Ar than the other species, and although not significant, *CG14073*, *Usp8*, and *cmb* showed lower expression in In than Ko and Es (Fig. 4). Therefore, these four genes may be involved in the formation of a long copulatory piece only in Ar. MPL8 contained a TF (*Mediator complex subunit 24* [*MED24*]) and a gene with cuticle development (*obstructor-A* [*obst-A*]). These two genes showed significant differences in expression profiles between species with long vs. short genital parts (Table S4). *MED24* and *obst-A* showed higher expression levels in Ar and In than Ko and Es in the PL stage, except for post hoc comparisons between In vs. Ko or Es (Fig. 4). Thus, high expressions of *MED24* and *obst-A* may be commonly involved in differences in copulatory piece length in these species.

In females, the FPE5 cluster contained no TF or gene with the GO term “imaginal disc development” or “cuticle development”, so we did not find candidate genes for differences in genital length. In the FPL1 cluster, we found 15 TFs, 48 genes involved in imaginal disc development, and 21 genes involved in cuticle development. Of these, 15 TFs, 44 genes for imaginal disc development, and 12 genes for cuticle development showed significant differences in the expression profiles between species with long and short vaginal appendices (Table S5). *Abd-B* was included in these genes (Fig. S5, Table S5). Three genes *dimmed* (*dimm*; TF), *Formin homology 2 domain containing* (*Fhos*; imaginal disc development), and *Cuticular protein 56F* (*Cpr56F*; cuticle development), showed the lowest FDRs. In the PL stage, these genes showed significantly lower expression in Ar and In than Ko and Es, and post hoc comparisons indicated significant differences between species with long vs. short genital parts (Fig. 4). Therefore, *dimm*, *Fhos*, and *Cpr56F* may be commonly involved in differences in the vaginal appendix length between species with long (Ar and Ko) and short genital parts (Ko and Es).

## Discussion

### Gene clusters associated with interspecific differences in genital length in each sex

**Fig. 5:**
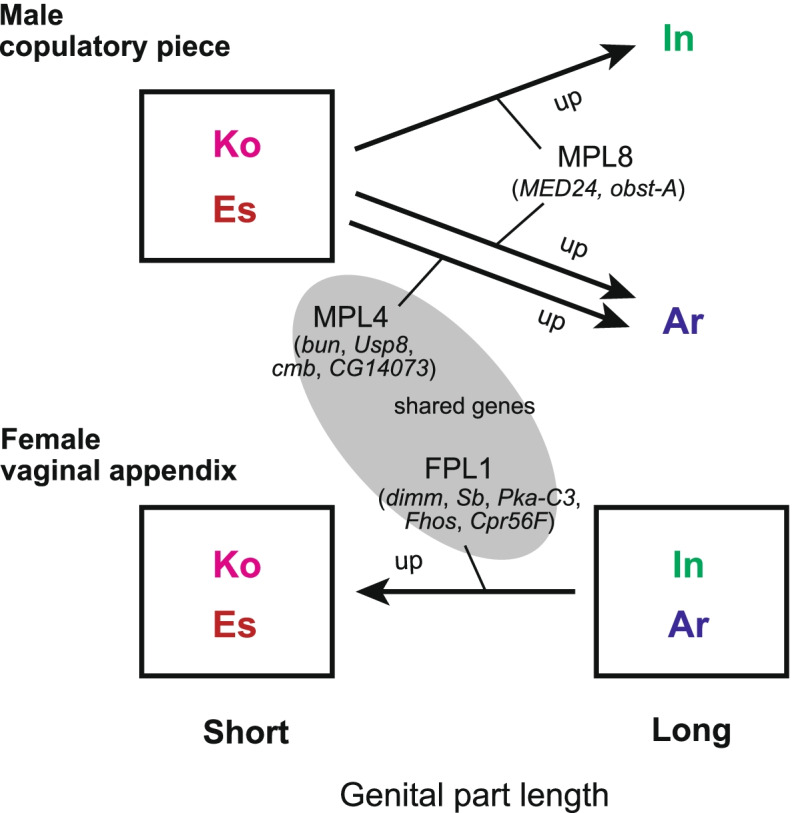
Summary of the expression changes in differences in genital part length implied by our results. Arrows indicate gene clusters for genital part length differences, and arrowheads indicate the species with upregulated gene expression. A shaded circle indicates that MPL4 and FPL1 contained shared genes. Species: Ar, *C*. *arrowianus*; In, *C*. *insulicola*; Ko, *C*. *komiyai*; and Es, *C*. *esakii*

We hypothesized that the common ancestor of the four species *C*. *arrowianus* (Ar), *C*. *insulicola* (In), *C*. *komiyai* (Ko), and *C*. *esakii* (Es) had long genital parts and that the common ancestor of Ko and Es had short, reduced genital parts. We also assumed that genes involved in differences in genital length would show similar expression profiles between two species with similar lengths but different expression profiles between species with different lengths. The MPL8 gene clusters in males and FPL1 in females fit the expected pattern, showing differences between species with long (Ar and In) and short (Ko and Es) parts, and contained genes possibly affecting genital length. In addition, the male MPL4 cluster, whose expression profile was similar only in species with short genital parts, was implicated in the interspecific difference in genital length based on the GO enrichment results. Among these clusters, no genes were shared between MPL8 and FPL1, but many were shared between FPL1 and MPL4. These results imply that female vaginal appendix length may be controlled by a single common gene expression mechanism (shifts in gene expression profiles in the FPL1 cluster), while male copulatory piece length may be controlled by common (shifts in gene expression profiles in the MPL8 cluster) and different mechanisms (shifts in gene expression profiles in the MPL4 cluster only for Ar) (Fig. 5). Thus, the results for male genital parts were partly inconsistent with our hypothesis, implying that different gene expression changes can lead to similar copulatory piece length even in closely related species. In addition, the changes in the expression profiles corresponding to the length of the copulatory piece and vaginal appendix differed between the sexes; genes in MPL4 and MPL8 showed lower expression levels in species with short compared to long copulatory pieces, whereas genes in FPL1 showed higher expression in species with short rather than long vaginal appendices (Figs. 2b and 3b). These results imply that the sex-discordant changes in gene expression profiles could be involved in the coevolution of male and female genital length, which may reflect differences in the regulation of gene expression and interspecific differences in genetic variation between the sexes. Thus, our study demonstrated that similar lengths of genitalia have been formed by different gene expression profiles in different species and sexes. However, the roles of the candidate genes in the genital development of *Ohomopterus* species are unknown and need to be clarified in future studies.

### Candidate genes related to differences in genital length and their expression profiles

We focused on the MPL8, MPL4, and FPL1 gene clusters and investigated candidate genes for the interspecies differences in genital part size based on the gene annotation information and comparisons of expression levels across species. In the male MPL8 cluster, the *MED24* transcription factor and the *obst-A* gene involved in cuticle development showed high expression in species with a long copulatory piece. *MED24* encodes a protein that regulates RNA polymerase II-dependent genes in *D. melanogaster* and is involved in pupal development and the regulation of programmed cell death [27]. *Obst-A* is involved in chitin-based cuticle development [28]. Genes involved in cuticle development may affect the morphology of species-specific copulatory pieces in other *Ohomopterus* species [15, 24], and *obst-A* may underlie the interspecific differences in copulatory piece length seen in our study species. Although *MED24* and *obst-A* are not known for their effects on genital morphology in *Drosophila* [29], these genes may be involved in differences in copulatory piece length between species with long (Ar and In) and short (Ko and Es) copulatory pieces.

In the MPL4 cluster, 41 genes were TFs and involved in imaginal disc and cuticle development (Table S4). This cluster included candidate genes related to species-specific genital morphology in three *Ohomopterus* species (*C*. *maiyasanus*, *C*. *iwawakianus*, and *C*. *uenoi*) with different genital sizes and morphologies, as seen in our previous study [24] (see Table S4). In MPL4, *bun* (TF) and *CG14073*, *Usp8*, and *cmb* (genes involved in imaginal disc development) were differentially expressed between Ar and Ko and between Ar and Es. *bun* is involved in organ development in *D*. *melanogaster* and affects wing and leg phenotypes [30]. *Usp8* is also involved in organ development through the hedgehog and wingless signalling pathways and affects wing phenotype [31]. These four genes (*bun*, *CG14073*, *Usp8*, and *cmb*) were highly expressed only in Ar and showed significantly different expression levels between Ar and Ko and between Ar and Es, but not between In and Ko or Es. Thus, the genes in the MPL4 cluster, particularly *bun* and *Usp8*, may be essential for forming the long copulatory piece in Ar but not in In. Therefore, different genes and gene expression may be used in forming long copulatory pieces in these species.

There were 63 genes in the FPL1 cluster in females, acting as TFs and genes involved in imaginal disc and cuticle development. These genes had different expression profiles between species with long and short vaginal appendices (Table S5). The FPL1 cluster also included candidate genes related to species-specific genital morphology in three *Ohomopterus* species (*C*. *maiyasanus*, *C*. *iwawakianus*, and *C*. *uenoi*), as seen in a previous study [24] (see Table S5). In FLP1, we focused on *dimm* (TF), *Fhos* (involved in imaginal disc development), and *Cpr56F* (involved in cuticle development), which showed different expression profiles between species with different vaginal appendix lengths. *dimm* regulates transcription by RNA polymerase II, is involved in organ development, and has a female-specific effect on wing phenotype in *D*. *melanogaster* [32], and may be involved in the formation of the female vaginal appendix in *Ohomopterus*. *Fhos* is involved in programmed cell death and affects the wing phenotype [33, 34]. Although it is unknown whether *Cpr56F* affects genital, leg, or wing phenotypes in *Drosophila*, this gene could be involved in the exaggeration of male genitalia in *C*. *uenoi* [24], and hence may also be involved in the formation of female genitalia in *Ohomopterus*. These genes showed low expression levels in In and Ar compared to Ko and Es in the PL stage. Therefore, the genes in the FPL1 cluster, but particularly *dimm*, *Fhos*, and *Cpr56F*, may be implicated in the differences between long and short vaginal appendices in the four species.

We identified some genes involved in species-specific genital parts among the three species of *Ohomopterus* among the TFs, genes involved in the imaginal disc, and the genes involved in cuticle development in the MPL4 and FPL1 [24]. In the MPL4 cluster (Table S4), we found three genes (*dumpy* [*dpy*], *spalt major* [*salm*], and *Cuticular protein 66D* [*Cpr66D*]) associated with differences in copulatory piece length in males of *C*. *iwawakianus* and *C*. *maiyasanus* that show similar differences in genital length as the four species compared in this study. Among these genes, *dpy* and *salm* showed high expression levels in *C*. *maiyasanus* with a long copulatory piece, while *Cpr66D* was highly expressed in *C*. *iwawakianus* with a short copulatory piece. This implies that some of the genes in MPL4 may be involved in the formation of the long copulatory pieces shared by *C*. *maiyasanus* and *C*. *arrowianus*. Because the QTL region for the genital differences in *C*. *iwawakianus* and *C*. *maiyasanus* showed a genomic divergence between *C*. *arrowianus* and *C*. *komiyai* [15], such divergent mutations might be involved in the expression of *dpy* and *salm*. However, because the genes in MPL4 showed low expression levels in *C*. *insulicola* with a long copulatory piece, it may be that not all species with similar copulatory piece lengths employ similar gene expression profiles.

In the FPL1 cluster (Table S5), three genes (*multiple wing hairs* [*mwh*], *CG42674*, and *methuselah-like 1* [*mthl1*]) were associated with differences in vaginal appendix length between *C*. *iwawakianus* and *C*. *maiyasanus*. These genes were highly expressed in *C*. *maiyasanus* with a long vaginal appendix, which differs from the patterns seen in this study, which showed low expression levels in species with a long vaginal appendix. This finding implies that the gene expression changes involved in different vaginal appendix length may differ between *C*. *iwawakianus*/*C*. *maiyasanus* and the four species in this study.

### Factors of genital coevolution and gene expression differences between the sexes

Our results support the hypothesis that a similar expression profile in a gene cluster (FPL1) likely controls the similar vaginal appendix length across the species in females. However, in males, both common (MPL8) and different (MPL4) gene clusters are likely involved in the differences in copulatory piece length among species. This difference between the sexes may be because the vaginal appendix is a membranous pocket with a simple and homogenous structure in the different species. By contrast, the copulatory pieces are sclerotized organs of variable shape and size among the different species. Indeed, despite the similarity in length, the elongated copulatory pieces of *C*. *arrowianus* and *C*. *insulicola* differ greatly in shape; in addition, the short copulatory pieces of *C*. *komiyai* and *C*. *esakii* differ greatly in shape and width (Fig. 1). Therefore, the evolution of genital part length in the genital coevolution between the sexes may have occurred through similar gene expression changes resulting in similar long or short lengths among species for the female vaginal appendix. By contrast, species-specific gene expression changes may have resulted in species-specific shapes with similar long or short lengths in the male copulatory pieces. The interspecific variation in copulatory piece morphology may have been affected by continuous selection pressure from species divergence to the present.

Although the female (FPL1) and male (MPL4) gene clusters shared many genes, their expression profiles in relation to the length of genital parts differed between the sexes. In a previous study of three *Ohomopterus* species with different-sized genital parts, including *C*. *iwawakianus* (short), *C*. *maiyasanus* (long), and *C*. *uenoi* (extremely long) [24], the genes and gene expression profiles affecting the differences in the genital parts between *C*. *iwawakianus* and *C*. *maiyasanus* differed between the sexes, which is consistent with the results presented here. On the other hand, there was sex-concordant gene expression in shared gene networks between the sexes involved in the extremely large differences in genital part length between *C*. *uenoi* and the other two species. Thus, previous results and those from the present study indicate that the gene expression profiles associated with genital part length differ between the sexes in species with short and long parts, and the sex-concordant gene expression in a shared gene network for the formation of extremely elongated genital parts of *C*. *uenoi* may be an exception.

Previous studies proposed that sexual conflict and reinforcement to avoid hybridization may have facilitated the coevolution of genital parts in *Ohomopterus* [11, 22, 35]. Takami et al. (2018) showed that a longer copulatory piece reduced female fitness but a longer vaginal appendix counteracted this [22]. Elongation of the copulatory piece could be facilitated by sperm competition [36], while the male and female genital parts are always subject to selection for matching to avoid damage to the genitalia and secure successful copulation and fertilization [11, 17]. Thus, the sexually antagonistic coevolution driven by sexual conflict must be under selection for matching. Fujisawa et al. [15] found that genetic loci controlling male and female genital length were located in different parts of the same linkage group in *C*. *iwawakianus* and *C*. *maiyasanus* and argued that coevolution between the sexes is only loosely constrained and can respond to sexually antagonistic evolution. Our gene expression profiles support the view that independent genetic control of genital size between the sexes facilitates sexually antagonistic coevolution. On the other hand, avoidance of maladaptive interspecific hybridization by the elongation or shortening of genital parts (reinforcement) is of common interest to both sexes. It can be achieved more rapidly by sex-concordant expression changes in a shared gene network, as was the case in *C*. *uenoi* [24]. Our results imply that the correlated evolution of male and female genital size via sexually antagonistic selection or the reinforcement selection to avoid maladaptive interspecific hybridization can be achieved by the same or different gene expression changes between species and between the sexes during and after species divergence.

## Conclusions

We explored changes in gene expression profiles in male and female genital tissues among four *Ohomopterus* species with different genital part lengths to understand the gene expression changes underlying the genital coevolution between the sexes. We found that species with similar genital part lengths showed similar expression levels for differentially expressed genes in females, but both common and different genes might be involved in the differences of genital length in males. Our study demonstrates that sex-independent and partly species-specific gene expression changes underlie the correlated evolution of male and female genital size. Thus, the genetic backgrounds underlying the genital coevolution between the sexes may be complicated. More detailed accounts for the responsible genes, their sequences, expression profiles, and roles involved in the diversification of genital size in *Ohomopterus* beetles are needed to fully understand the coevolutionary dynamics of the functionally corresponding male and female genital morphology.

## Methods

### Sampling for genome assembly and transcriptome study

We collected adult *Carabus* (*Ohomopterus*) *insulicola* at Funato, Kashiwa, Chiba; *C*. *arrowianus* at Miyakoda Park Complex, Hamamatsu, Shizuoka; *C*. *komiyai* at Mt. Ogasa, Kakegawa, Shizuoka; and *C*. *esakii* at Mt. Takakusa, Yaizu, Shizuoka from May–June 2018. A *C*. *esakii* male was fixed in 99% EtOH and stored at -30℃ until DNA extraction for draft genome assembly. Live adult beetles of four species were reared at 20 °C under long-day conditions (light: dark [LD], 16:8 h), and eggs deposited by females were raised to pupae, which were used for the transcriptome analyses. Details of breeding methods have been described previously [24, 37]. We fixed the pupae in RNAlater solution (Invitrogen, Carlsbad, CA, USA) at two stages: the early pupal stage (PE) at 1–3 days after pupation, and the late pupal stage (PL) at 4–6 days after pupation. We obtained three samples from each stage for males and females; the sex of the pupae was identified by the external morphology of the abdominal tips, such that the apical part of the aedeagus protrudes from the tip of the abdomen in males and the entire aedeagus becomes visible as the pupal period progresses (Table S2).

### DNA extraction and sequencing for the draft genome

We assembled a draft genome of *Carabus esakii* using the 10 × Genomics Chromium Linked-Reads (10 × Genomics) as a reference genome for transcriptome analyses of the four study species. Total genomic DNA was extracted from testes of a *C*. *esakii* male using a QIAGEN Genomic-tip 20/G with Blood & Cell Culture DNA Kits (Qiagen, Hilden, Germany). The extracted genomic DNA was used for library construction of 150 bp paired-end 10 × Genomics Chromium Linked-Reads. Sequence library construction and sequencing with an Illumina Hiseq X Ten platform were performed by the Beijing Genomic Institute (BGI) (Table S1).

### RNA extraction and sequencing for transcriptome analyses

We extracted total RNA from the genital parts of pupae (*n* = 3 for each sex of each species) according to our previous studies [24, 37]. Messenger RNA (mRNA) was isolated from total RNA using NEBNext Poly(A) mRNA Magnetic Isolation Module (New England Biolabs, Ipswich, MA, USA), and RNA-seq libraries were constructed from isolated mRNA using YourSeq Duet RNAseq Library Kits (Amaryllis Nucleics, Oakland, CA, USA), following the manufacturer’s instructions. Library preparations for 150 bp paired-end sequence reads were made using KAPA Library Preparation Kits for Illumina (Kapa Biosystems, Wilmington, MA, USA). Libraries were sequenced using the Illumina HiSeq X Ten platform at Macrogen Co. Ltd. (Kyoto, Japan). The library construction and sequencing were performed in three batches (Table S2).

### Draft genome assembly and gene prediction

We reconstructed the *C*. *esakii* draft genome from 10 × Genomics Chromium linked reads using Supernova v. 2.1.1 [38]. Prior to the genome assembly, we estimated the genome size with the linked reads by a *k*-mer spectrum analysis using Jellyfish 2.2.10 [39] and GenomeScope [40]; the estimated genome size was 184 Mbp (Fig. S6), comparable to that of closely related *C. uenoi*, 186 Mbp [15]. In genome assembly, we used 746,666 linked reads equivalent to 56-fold coverage of an approximate genome size of 200 Mbp according to the protocol of Supernova (Table S1). The completeness of the assembled genome was assessed using BUSCO v. 4.1.2 [41] with the Endopterygota orthologs (endopterygota_odb10). Repeat sequences on the assembled scaffolds were identified and masked using RepeatModeler v. 2.0.1 and RepeatMasker v. 4.0.9 [42]. Using the masked *C*. *esakii* genome, we performed gene prediction using Braker2 pipeline v. 2.1.4 [43–50]. Hint files for gene prediction were generated by mapping *C*. *esakii* transcriptome data to the draft genome using Hisat2 v. 2.1.0 [51]. The protein-coding regions of the predicted gene sets were matched to *Drosophila melanogaster* RefSeq proteins using BLASTX (*E*-value < 1e-5) [52, 53] to obtain orthology information.

### Quality control of the RNA sequence data and read counts

Prior to the transcriptome analyses, we trimmed the RNA-seq reads using fastp v. 0.20.0 [54]. We used the default parameters except for the removal of the poly X sequence at the 3’ ends, and the minimum read length after trimming was set to 25. The trimmed reads were mapped to the predicted exon regions in the draft *C*. *esakii* genome using the paired-end option in STAR v. 2.7.5c [55]. We mapped RNA-seq reads only on draft genome scaffolds longer than 10 kbp to prevent mapping on genes that were fragmented between scaffolds. The mapping rates of reads were comparable among four species (Table S2). To estimate gene expression levels, mapped RNA-seq reads were counted using featureCounts v. 1.5.1 [56]. Read count data for genes with average read counts per sample < 10 among all of the pupal samples were excluded from the following analyses, as they were considered noise (13,989 of 23,767 genes).

### Gene expression profiles and variance analyses

We performed PCA on the 48 samples of *C*. *arrowianus*, *C. insulicola*, *C*. *komiyai*, and *C*. *esakii* data to summarize the variation in gene expression profiles among the samples using the prcomp function in R [57]. Prior to PCA, we normalized read counts using the TCC v. 1.14.0 [58] package in R and converted the normalized read counts to *Z*-scores. Scores obtained for PC1 and PC2 were tested using a linear mixed model (lmer function in R) to investigate fixed effects of species, sex, or stage differences and a random effect of sequencing batch difference. We confirmed that the batch effect was absent (Table S3).

We evaluated the contributions of developmental stage (PE, PL), species, and sex to the obtained gene expression variance using a linear model implemented in the variancePartition v. 1.18.3 package in R [59]. All variables were modelled as random effects because they were categorical. The linear model was as follows:

$$\mathrm{Gene\,expression }\sim \left(1|\mathrm{Stages}\right)+\left(1|\mathrm{Species}\right)+\left(1|\mathrm{Sex}\right)$$

### Differentially expressed genes between species

We identified DEGs among four species in each stage and sex using the DESeq2 v. 1.14.1 [60] package in R [57]. We clustered the DEGs according to the expression profiles using a hierarchical clustering method with the hclust function in R to classify the DEGs according to the different profile expression patterns among the species for each stage and sex. We defined clusters for candidate gene searches as those separated by distances (height) > 3 from one another (Figs. S3, S4). We normalized the read counts using the TCC v. 1.14.0 package in R [58] and converted the normalized read counts to Z-scores before the clustering analyses. We identified similarities in the expression profiles of the genes included in each cluster using pvclust v. 2.2–0 [61] in R. For each cluster, GO enrichment analysis was performed using Metascape [62], and terms with a FDR < 0.01 were identified as the functions of the genes included in the cluster. Among the GO terms, we focused on “imaginal disc development”, “genital disc development” (within “imaginal disc development”), and “cuticle development”. Furthermore, we investigated the candidate genes involved in interspecific differences in genital part size in the clusters selected based on their expression profiles or GO enrichment results. The candidate genes were inferred by genes that were classified as TFs by the UniProtKB [63] database or with the GO terms “imaginal disc development” and “cuticle development” by the FlyBase database [29]. The effects of genital part length and species on expression levels of the candidate genes were examined using a nested ANOVA. We calculated the FDR in the effects of genital length and species using nested ANOVA with the qvalue package in R [64]. We tested differences in the expression of the candidate genes among species for each stage using Tukey’s honestly significant difference test for statistical significance.

## Supplementary Information

**Additional file 1.**

## Data Availability

All raw read data and draft genome of *C. esakii* have been deposited at DNA Data Bank of Japan Sequence Read Archive (BioProject PRJDB5403; Table S1, S2). Draft genome of *C. esakii* have been deposited at DNA Data Bank of Japan Annotated/Assembled Sequences (BQUW01000001-BQUW01012194). An annotated gene list with expression data are archived at figshare (https://doi.org/10.6084/m9.figshare.c.5868749.v1).

## Abbreviations

DNA: Deoxyribonucleic acid
RNA: Ribonucleic acid
QTL: Quantitative trait locus
Ar: *Carabus arrowianus*
In: *Carabus insulicola*
Ko: *Carabus komiyai*;
Es: *Carabus esakii*
DEG: Differentially expressed gene
GO: Gene ontology
PE: Pupa early stage
PL: Pupa late stage
PCA: Principle component analysis
PC1: Principle component score 1
PC2: Principle component score 2
FDR: False discovery rate
MPE: Clusters in the male PE stage
MPL: Clusters in the male PL stage
FPE: Clusters in the female PE stage
FPL: Clusters in the female PL stage
*Abd-B*: *Abdominal-B*
*dsx*: *doublesex*
TF: Transcription factor
*bun*: *bunched*
*Usp8*: *Ubiquitin specific protease 8*
*cmb*: *combover*
*MED24*: *Mediator complex subunit 24*
*obst-A*: *obstructor-A*
*dimm*: *dimmed*
*Fhos*: *Formin homology 2 domain containing*
*Cpr56F*: *Cuticular protein 56F*
*dpy*: *dumpy*
*salm*: *spalt major*
*Cpr66D*: *Cuticular protein 66D*
*mwh*: *multiple wing hairs*
*mthl1*: *me* *thus* *elah-like 1*
EtOH: Ethanol
mRNA: Messenger RNA
RNA-seq: RNA sequencing
ANOVA: Analysis of variance
