# Supplementary material for: Evolutionary changes in gene expression profiles associated with the coevolution of male and female genital parts among closely related ground beetle species
Source: BMC Genomics. 2022 Sep 8;23:637. doi: 10.1186/s12864-022-08865-2 (PMC9454128; doi:10.1186/s12864-022-08865-2)
Supplement: Supplementary file 1 — Additional file 1. [file 12864_2022_8865_MOESM1_ESM.pdf]

Evolutionary changes in gene expression profiles associated with the coevolution of male and female genital parts among closely related ground beetle species

Shota Nomura[1,2] and Teiji Sota[1]

[1]Department of Zoology, Graduate School of Science, Kyoto University, Sakyo, Kyoto, 606-8502, Japan.
[2]Division of Evolutionary Developmental Biology, National Institute for Basic Biology, 38, Nishigonaka, Myodaiji, Okazaki, 444- 8585, Japan

Supplementary information

Fig. S1—Fig. S6.
Table S1—Table S5

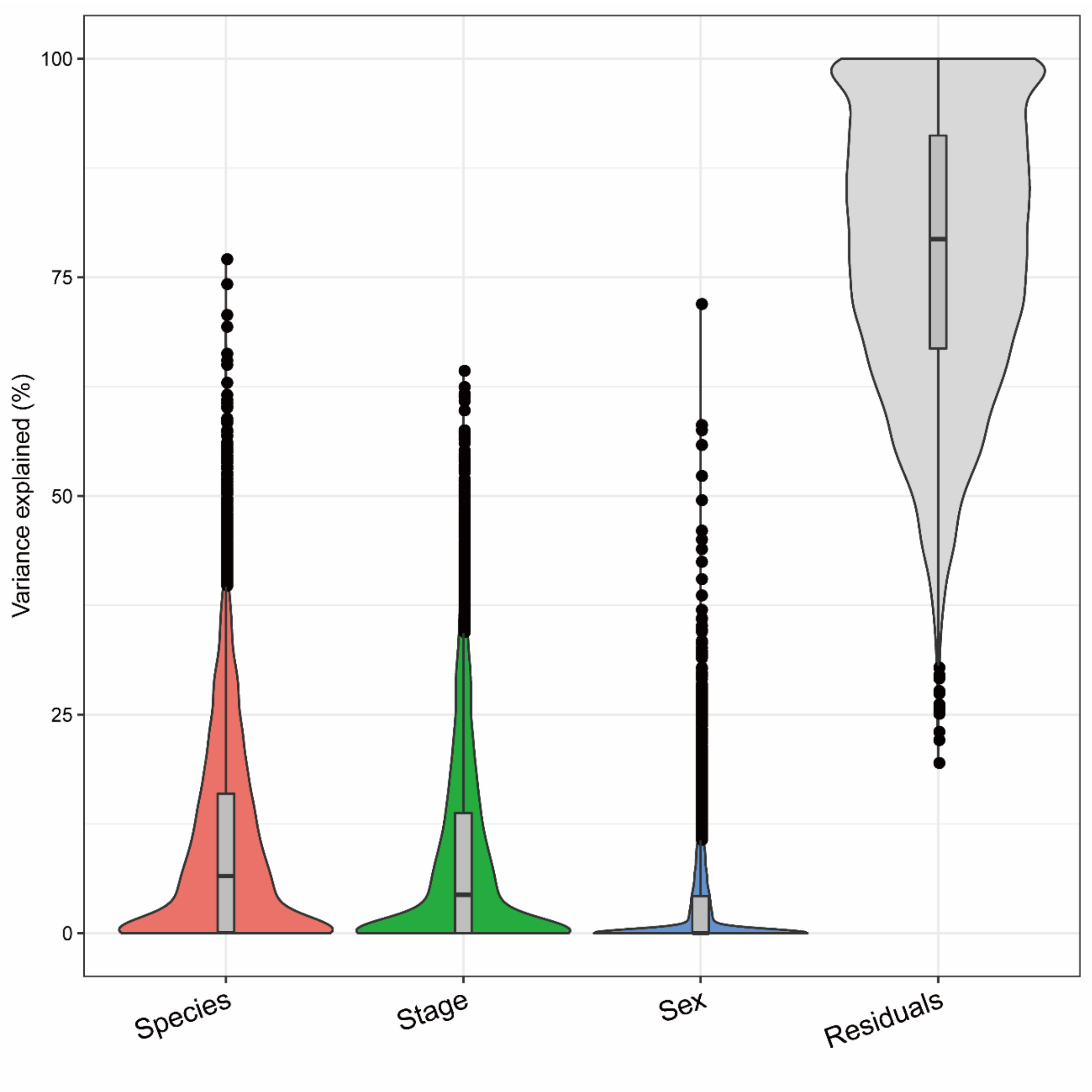

Fig. S1. Percentages of the variance in gene expression explained by stage, species, and sex differences for all 9,778 genes in the four species.

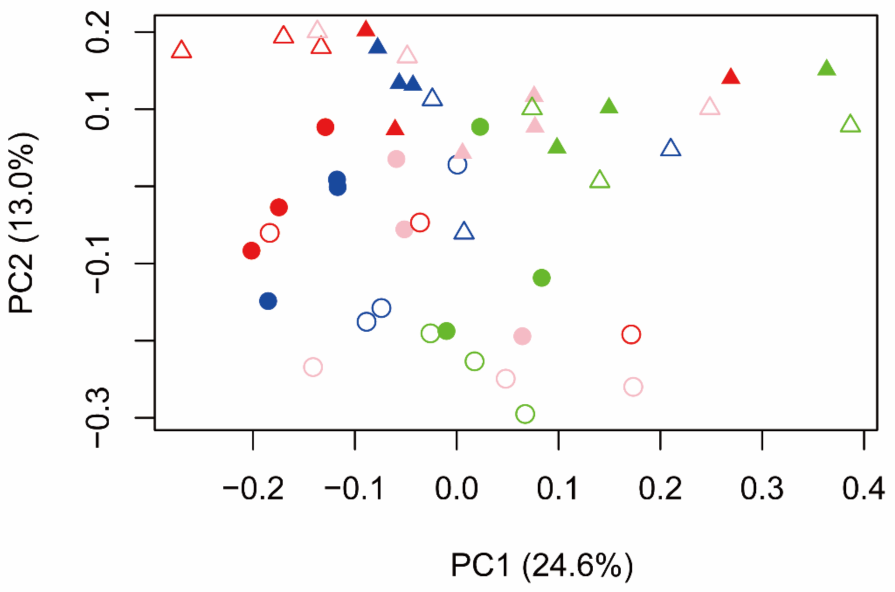

Fig. S2. The relationships between the first and second principal component scores (PC1 vs. PC2) resulting from the principal component analysis with read counts data from individual samples. The proportion of variance explained by each principal component is given in parentheses. Filled circle, male early-stage pupae (PE); open circle, female PE; filled triangle, male late-stage pupae (PL); open triangle, female PL; blue, *C. arrowianus*; green, *C. insulicola*; pink, *C.komiyai*; red, *C. esakii*.

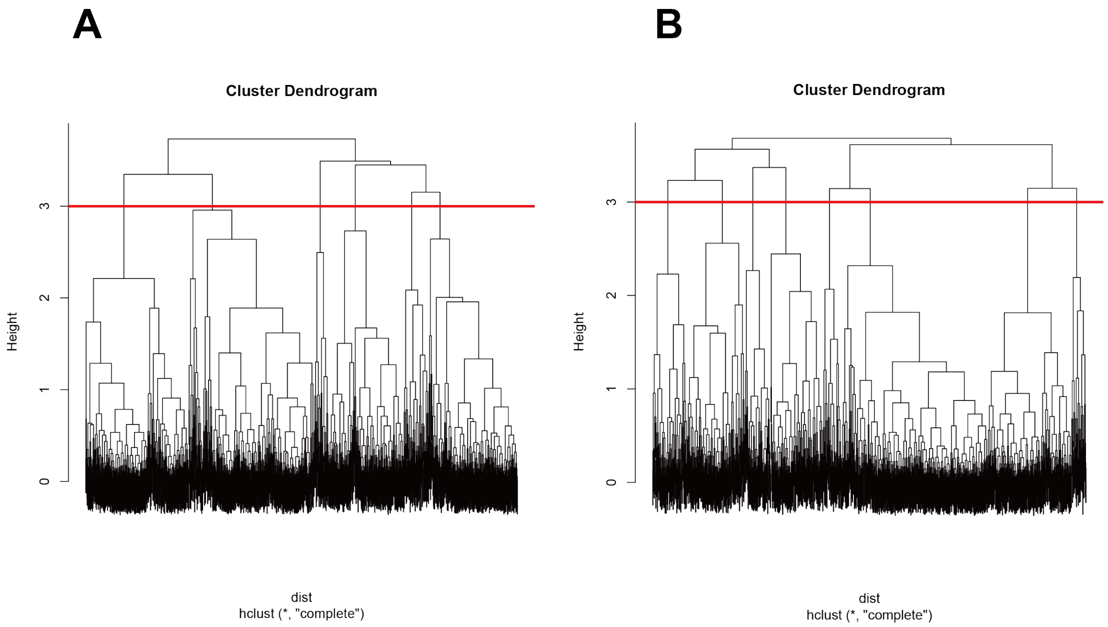

Fig. S3. Clustering of DEGs based on expression levels in males of the PE stage (*A*) and the PL stage (*B*). DEGs were divided into different clusters at height = 3.

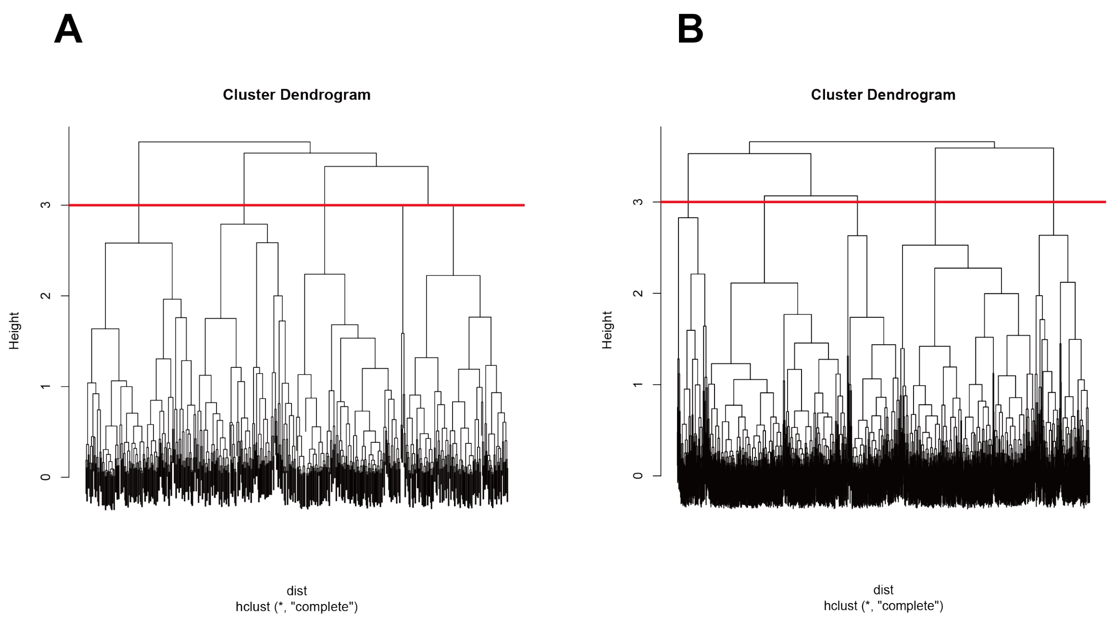

Fig. S4. Clustering of DEGs based on expression levels in females of the PE stage (*A*) and the PL stage (*B*). DEGs were divided into different clusters at height = 3.

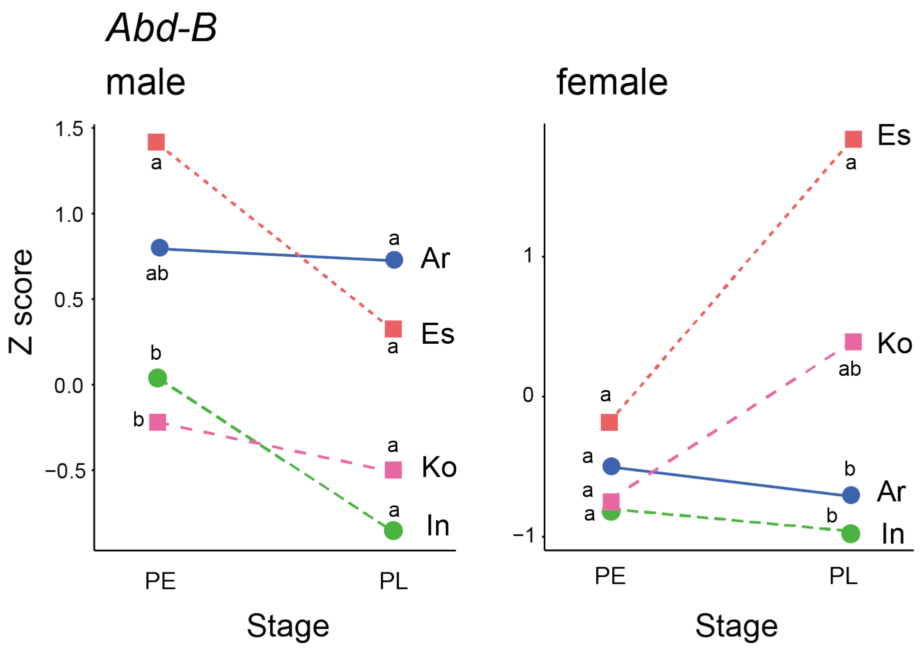

Fig. S5. Interspecific differences in the expression profiles of the *Abd-B* gene. Species: Ar, *C. arrowianus*; In, *C. insulicola*; Ko, *C. komiyai*; and Es, *C. esakii*. Expression levels with the same letter (a, b, c) are not significantly different from one another ($P > 0.05$) among species in each stage by the multiple comparison test.

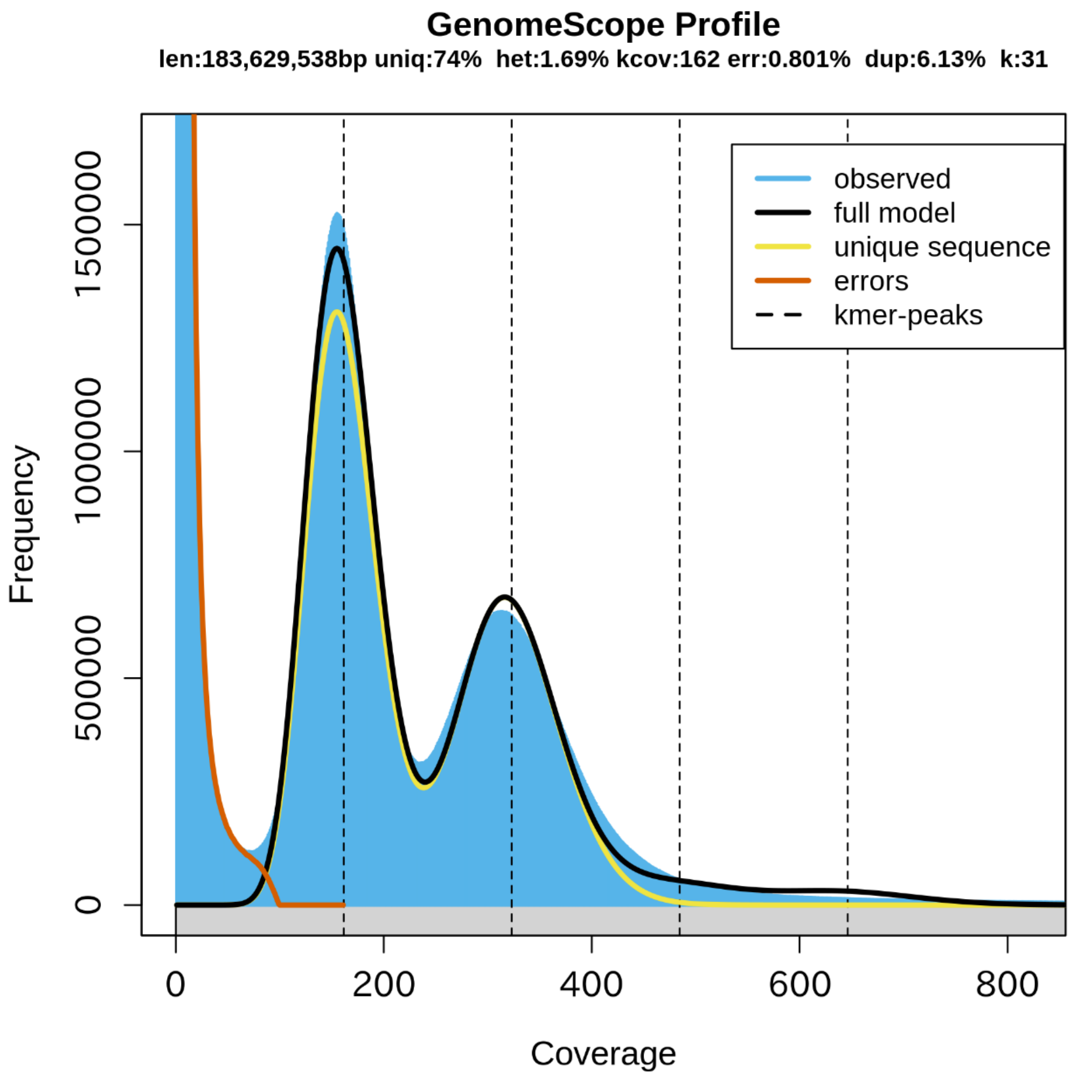

Fig. S6. The *k*-mer spectrum profile of 10x Genomics Chromium linked reads for *C. esakii* (*k* = 31) generated by GenomeScope. The estimated genome size was 183.6 Mbp.

Table S1. Details of DNA samples and read data used for the whole genome assembly and the 10x Genomics Chromium linked reads.

Species	*Carabus esakii*
Sex	male
Tissue	testit
Collection site	Mt. Takakusa, Yaizu city, Shizuoka
DDBJ BioProject ID	PRJDB5403
DDBJ DRR Run accession number	DRR346635
Total raw reads	720,106,184
Read length	150
Q20 (%)	95.39
GC (%)	38.31
Total read pairs used for assembly	746,666

Table S2. Details of RNA-seq samples and read data used in the gene expression profiling for *Carabus arrowianus* , *C. esakii* , *C. insulicola* , and *C. komiyai* pupae.

						**Raw read metrics**		**Mapped read metrics**		
Sample	Stage1	Species	Sex	Sequence platform	Batch	Raw read pairs	Average phred score	Mapped read	Mapping rate(%)	DDBJ DRR Run accession number2
ArPE1m	PE	*C. arrowianus*	male	HiSeqX	B	10917654	39	7404884	81.3	DRR345708
ArPE2m	PE	*C. arrowianus*	male	HiSeqX	B	13939316	39	9091944	79.0	DRR345709
ArPE3m	PE	*C. arrowianus*	male	HiSeqX	B	12195805	39	8088364	82.0	DRR345710
ArPE4f	PE	*C. arrowianus*	female	HiSeqX	B, C	15595219	38	9901013	76.2	DRR345711
ArPE5f	PE	*C. arrowianus*	female	HiSeqX	B	9341991	39	6324137	81.1	DRR345712
ArPE6f	PE	*C. arrowianus*	female	HiSeqX	B, C	12555099	39	7793862	76.5	DRR345713
ArPL1m	PL	*C. arrowianus*	male	HiSeqX	A	14736722	39	10608202	82.2	DRR345714
ArPL2m	PL	*C. arrowianus*	male	HiSeqX	A	11754220	39	8468706	80.5	DRR345715
ArPL3m	PL	*C. arrowianus*	male	HiSeqX	A, B	13634397	39	9889396	81.6	DRR345716
ArPL4f	PL	*C. arrowianus*	female	HiSeqX	B	8298580	39	9944921	79.5	DRR345717
ArPL5f	PL	*C. arrowianus*	female	HiSeqX	B	12982946	39	8082549	75.3	DRR345718
ArPL6f	PL	*C. arrowianus*	female	HiSeqX	B	12033014	39	7864767	78.6	DRR345719
EsPE1m	PE	*C. esakii*	male	HiSeqX	B	10241344	39	6556070	78.3	DRR345720
EsPE2m	PE	*C. esakii*	male	HiSeqX	B, C	14100144	39	9333561	79.0	DRR345721
EsPE3m	PE	*C. esakii*	male	HiSeqX	B	19936823	39	14184724	83.0	DRR345722
EsPE4f	PE	*C. esakii*	female	HiSeqX	B	9532813	39	5876507	71.8	DRR345723
EsPE5f	PE	*C. esakii*	female	HiSeqX	B	9665941	39	6793526	81.2	DRR345724
EsPE6f	PE	*C. esakii*	female	HiSeqX	C	27071470	39	20388152	85.8	DRR345725
EsPL1m	PL	*C. esakii*	male	HiSeqX	B, C	12833777	39	9403655	81.4	DRR345726
EsPL2m	PL	*C. esakii*	male	HiSeqX	B, C	15116804	39	10719157	81.0	DRR345727
EsPL3m	PL	*C. esakii*	male	HiSeqX	B	12732132	39	9247475	83.7	DRR345728
EsPL4f	PL	*C. esakii*	female	HiSeqX	B	18705944	39	12962278	82.2	DRR345729
EsPL5f	PL	*C. esakii*	female	HiSeqX	B	17364818	39	11656613	80.8	DRR345730
EsPL6f	PL	*C. esakii*	female	HiSeqX	B	12883178	39	9496193	84.5	DRR345731
InPE1m	PE	*C. insulicola*	male	HiSeqX	A	11767184	39	8213384	79.4	DRR345732
InPE2m	PE	*C. insulicola*	male	HiSeqX	A	15566724	39	9939483	74.1	DRR345733
InPE3m	PE	*C. insulicola*	male	HiSeqX	A, B, C	10786637	39	7495784	78.3	DRR345734
InPE4f	PE	*C. insulicola*	female	HiSeqX	A, B	11434403	39	6971897	72.9	DRR345735
InPE5f	PE	*C. insulicola*	female	HiSeqX	A, B	8712357	39	5750418	75.8	DRR345736
InPE6f	PE	*C. insulicola*	female	HiSeqX	A, B	11407182	39	7826091	77.9	DRR345737

InPL1m	PL	*C. insulicola*	male	HiSeqX	A, B	8768670	39	4910380	63.4	DRR345738
InPL2m	PL	*C. insulicola*	male	HiSeqX	A, B	10493660	39	7227984	78.9	DRR345739
InPL3m	PL	*C. insulicola*	male	HiSeqX	A, B, C	21374330	39	16057807	83.3	DRR345740
InPL4f	PL	*C. insulicola*	female	HiSeqX	A, B	9816612	39	6864752	81.1	DRR345741
InPL5f	PL	*C. insulicola*	female	HiSeqX	A	18532943	39	12328492	72.4	DRR345742
InPL6f	PL	*C. insulicola*	female	HiSeqX	A, B, C	9619381	39	6793344	78.4	DRR345743
KoPE1m	PE	*C. komiyai*	male	HiSeqX	A	11894446	39	8611287	81.2	DRR345744
KoPE2m	PE	*C. komiyai*	male	HiSeqX	A, B	12116693	39	9076033	82.1	DRR345745
KoPE3m	PE	*C. komiyai*	male	HiSeqX	A	12095394	39	8090553	75.7	DRR345746
KoPE4f	PE	*C. komiyai*	female	HiSeqX	A	10677335	39	7533208	78.4	DRR345747
KoPE5f	PE	*C. komiyai*	female	HiSeqX	A	13033357	39	8676816	72.1	DRR345748
KoPE6f	PE	*C. komiyai*	female	HiSeqX	B	14745716	39	9400841	78.9	DRR345749
KoPL1m	PL	*C. komiyai*	male	HiSeqX	A, B	16394567	39	12512752	82.7	DRR345750
KoPL2m	PL	*C. komiyai*	male	HiSeqX	A	9485040	39	7259221	83.1	DRR345751
KoPL3m	PL	*C. komiyai*	male	HiSeqX	A	12532274	39	9408127	81.8	DRR345752
KoPL4f	PL	*C. komiyai*	female	HiSeqX	A	13376670	39	10193814	85.5	DRR345753
KoPL5f	PL	*C. komiyai*	female	HiSeqX	A	16466667	39	12091657	84.7	DRR345754
KoPL6f	PL	*C. komiyai*	female	HiSeqX	A	12698470	39	9300917	79.7	DRR345755

1) PE, early pupa; PL, late pupa.

2) BioProject ID: PRJDB5403.

Table S3. Fixed effects of species, stage, and sex differences and a random effect of sequencing batch differences on principal component scores (PC1 and PC2) which were summarized the variation of gene expression profiles among samples of *C. arrowianus* (Ar) , *C. esakii* (Es), *C.insulicola* (In), and *C. komiyai* (Ko).

Fixed effects

	Estimate	Std.	*t*	*P*
PC1				
Intercept	-0.08182	0.04371	-1.872	0.06818
Species (Es)	-0.03686	0.05047	-0.730	0.46923
Species (In)	0.16108	0.05047	3.192	0.00268
Species (Ko)	0.06843	0.05047	1.356	0.18241
Stage (PL)	0.08299	0.03569	2.325	0.02495
Sex (Male)	-0.01567	0.03569	-0.439	0.66285
PC2				
Intercept	-0.13689	0.02922	-4.685	2.94E-05
Species (Es)	0.04441	0.03374	1.316	0.1953
Species (In)	-0.04589	0.03374	-1.360	0.1811
Species (Ko)	-0.02911	0.03374	-0.863	0.3932
Stage (PL)	0.22500	0.02386	9.430	6.27E-12
Sex (Male)	0.06407	0.02386	2.685	0.0103

Random effect

	Variance	SD
PC1		
Batch	0.0000	0.0000
Residual	0.0153	0.1236
PC2		
Batch	0.0000	0.0000
Residual	0.0068	0.0827

Table S4. Effects of copulatory piece lengths (CPL; short vs. long) and species on the expression levels of the genes with GO terms "imaginal disc development", "cuticle development" and transcription factors in MPL4 and MPL8 clusters.

					Hub genes in Nomura et al. (2021)			
					IvM		UvIM	
Gene ID	Abbreviation	Gene name	FDR-*P* (CPL)	FDR-*P* (CPL:Species)	male	female	male	female
MPL4 cluster								
g12088	CG14073	CG14073	9.96E-01	**1.29E-02**				
g6294	Smr	smrter	9.91E-01	**1.29E-02**				
g3726	bun	bunched	4.89E-01	**1.29E-02**				
g17513	Usp8	ubiquitin specific protease 8	8.63E-01	**1.29E-02**				
g18367	cmb	combover	8.63E-01	**2.15E-02**				
g1328	Wnk	Wnk kinase	8.63E-01	**2.15E-02**				
g4709	kto	kohtalo	9.91E-01	**3.39E-02**				
g17072	ksr	kinase suppressor of ras	8.63E-01	**3.39E-02**				
g17175	zfh2	Zn finger homeodomain 2	9.96E-01	**3.64E-02**				
g11716	mthl1	methuselah-like 1	9.99E-01	**3.64E-02**		○	○	○
g1651	Hipk	homeodomain interacting protein kinase	9.96E-01	**3.69E-02**				
g1258	Ptip	PAX transcription activation domain interacting protein	9.99E-01	**3.69E-02**				
g13724	knk	knickkopf	8.63E-01	**3.82E-02**				
g454	Pka-C1	protein kinase, cAMP-dependent, catalytic subunit 1	9.96E-01	**4.28E-02**				
g9772	wge	winged eye	9.96E-01	**4.41E-02**				
g5222	ck	crinkled	8.63E-01	**4.41E-02**				
g5989	skd	skuld	9.96E-01	**4.41E-02**				
g12776	kkv	krotzkopf verkehrt	8.63E-01	**4.46E-02**				
g13456	dpy	dumpy	8.63E-01	**4.73E-02**	○		○	
g3591	CG8405	CG8405	9.96E-01	**4.80E-02**				
g3778	sbb	scribbler	9.99E-01	**4.83E-02**				
g14158	RhoGEF2	Rho guanine nucleotide exchange factor 2	9.99E-01	**4.83E-02**				
g19071	Exn	ephexin	9.99E-01	**4.83E-02**				
g1309	Gug	grunge	9.96E-01	**4.83E-02**				
g3905	bbg	big bang	8.63E-01	**4.97E-02**				
g7608	Bap170	brahma associated protein 170kD	9.96E-01	6.29E-02				
g7657	ds	dachsous	9.91E-01	6.38E-02				
g15516	mgl	megalin	9.91E-01	6.55E-02		○		
g15495	JIL-1	JIL-1 kinase	9.99E-01	7.31E-02				
g4968	salm	spalt major	9.99E-01	8.56E-02	○		○	
g4865	Cpr66Cb	cuticular protein 66Cb	9.91E-01	9.67E-02				
g5320	Cpr66D	cuticular protein 66D	8.63E-01	9.67E-02	○			
g1270	scaf	scarface	8.63E-01	9.78E-02				
g13665	stw	straw	8.63E-01	1.34E-01		○	○	
g15960	m	miniature	9.96E-01	1.62E-01				
g5509	CG42674	CG42674	9.96E-01	1.62E-01		○		
g15383	Cpr56F	cuticular protein 56F	8.63E-01	1.62E-01			○	
g317	Cpr100A	cuticular protein 100A	8.63E-01	2.20E-01				
g5059	Cpr65Az	cuticular protein 65Az	8.63E-01	2.50E-01				
g5360	Cpr65Av	cuticular protein 65Av	8.63E-01	3.63E-01				
g13589	Nos	nitric oxide synthase	9.91E-01	3.76E-01				
MPL8 cluster								
g11876	obst-A	obstructor-A	**1.91E-03**	2.59E-01				
g5959	MED24	mediator complex subunit 24	**2.90E-03**	6.99E-01				

Table S5. Effects of vaginal appendix lengths (VAL; short vs. long) and species on the expression levels of the genes with GO terms "imaginal disc development", "cuticle development" and transcription factors in FPL1 clusters.

					Hub genes in Nomura et al. (2021)			
					IvM		UvIM	
Gene ID	Abbreviation	protein	FDR-*P* (VAL)	FDR-*P* (VAL:Species)	male	female	male	female
g14604	Fhos	formin homology 2 domain containing	**4.57E-04**	2.68E-01				
g15383	Cpr56F	cuticular protein 56F	**2.20E-03**	4.91E-01			○	
g3888	mwh	multiple wing hairs	**2.20E-03**	2.68E-01		○		
g9762	dimm	dimmed	**2.20E-03**	9.96E-01				
g992	cora	coracle	**6.06E-03**	9.17E-01				
g7044	Dp	DP transcription factor	**6.06E-03**	2.68E-01				
g9881	f	forked	**6.06E-03**	9.66E-01				
g3668	lig	lingerer	**6.06E-03**	6.80E-01				
g12292	Sb	stubble	**6.06E-03**	9.96E-01				
g13011	Pka-C3	protein kinase, cAMP-dependent, catalytic subunit 3	**6.47E-03**	9.66E-01				○
g9451	psq	pipsqueak	**6.47E-03**	9.17E-01			○	
g38	Hr3	hormone receptor 3	**7.74E-03**	4.14E-01				
g5661	Mad	mothers against dpp	**7.74E-03**	2.68E-01				
g13761	RhoGAP19D	Rho GTPase activating protein at 19D	**7.74E-03**	2.68E-01				
g6294	Smr	smrter	**7.74E-03**	6.38E-01				
g9772	wge	winged eye	**7.74E-03**	3.84E-01				
g10436	Hr4	hormone receptor 4	**8.42E-03**	2.81E-01			○	
g5509	CG42674	CG42674	**8.42E-03**	9.96E-01		○		
g1826	crm	cramped	**8.42E-03**	3.41E-01				
g5222	ck	crinkled	**8.42E-03**	9.66E-01				
g12528	crb	crumbs	**8.42E-03**	2.68E-01				
g17608	CrebA	Cyclic-AMP response element binding protein A	**8.42E-03**	2.81E-01				
g19487	RhoGEF64C	Rho guanine nucleotide exchange factor at 64C	**8.42E-03**	9.90E-01				
g3414	sima	similar	**8.42E-03**	3.87E-01				
g12335	scrib	scribbled	**8.45E-03**	6.17E-01				
g11716	mthl1	methuselah-like 1	**8.84E-03**	6.17E-01		○	○	○
g15014	Abd-B	abdominal B	**8.84E-03**	4.52E-01				
g19360	H	hairless	**9.69E-03**	2.68E-01				
g12088	CG14073	CG14073	**9.93E-03**	9.66E-01				
g14601	Fkbp14	FK506-binding protein 14	**9.93E-03**	9.66E-01				
g6900	pcm	pacman	**9.93E-03**	2.68E-01				
g5304	tai	taiman	**9.93E-03**	2.68E-01				
g6724	fbl	fumble	**1.12E-02**	6.17E-01				
g1651	Hipk	homeodomain interacting protein kinase	**1.12E-02**	2.81E-01				
g3778	sbb	scribbler	**1.12E-02**	3.89E-01				
g11321	Ada2b	transcriptional adaptor 2b	**1.12E-02**	3.89E-01				
g3591	CG8405	CG8405	**1.17E-02**	8.74E-01				
g13516	Cyp18a1	cytochrome P450-18a1	**1.17E-02**	9.66E-01				
g14602	Fhos	formin homology 2 domain containing	**1.17E-02**	9.66E-01				
g17769	Nrg	neuroglian	**1.17E-02**	2.68E-01				
g17783	dsx-c73A	doublesex cognate 73A	**1.23E-02**	9.96E-01				
g7671	ds	dachsous	**1.35E-02**	3.41E-01				
g19694	ect	ectodermal	**1.35E-02**	8.25E-01				
g10924	fru	fruitless	**1.35E-02**	9.66E-01				
g15495	JIL-1	JIL-1 kinase	**1.35E-02**	4.42E-01				
g18512	lilli	lilliputian	**1.35E-02**	4.43E-01				
g10927	fru	fruitless	**1.47E-02**	4.91E-01				
g13961	Syx1A	syntaxin 1A	**1.61E-02**	9.66E-01				
g5161	ttk	tramtrack	**1.61E-02**	2.81E-01				
g1328	Wnk	Wnk kinase	**1.92E-02**	9.96E-01				
g17685	Alas	aminolevulinate synthase	**2.01E-02**	9.90E-01				
g14158	RhoGEF2	Rho guanine nucleotide exchange factor 2	**2.08E-02**	5.71E-01				
g14159	RhoGEF2	Rho guanine nucleotide exchange factor 2	**2.08E-02**	4.91E-01				
g3121	Gld	glucose dehydrogenase	**2.13E-02**	9.66E-01				
g10750	cv-d	crossveinless d	**2.21E-02**	8.74E-01				
g4968	salm	spalt major	**2.31E-02**	3.85E-01	○		○	
g21184	fw	furrowed	**2.37E-02**	9.17E-01			○	
g3496	Poxn	Pox neuro	**2.67E-02**	8.74E-01				
g18561	sdt	stardust	**2.80E-02**	7.00E-01				
g21109	tok	tolkin	**3.42E-02**	9.66E-01				
g13664	stw	straw	**3.45E-02**	9.66E-01			○	
g12776	kkv	krotzkopf verkehrt	**4.33E-02**	7.68E-01				
g5172	Spn88Ea	serpin 88Ea	**4.52E-02**	9.66E-01				
g3493	Vajk4	Vajk4	5.07E-02	9.96E-01				
g21251	Cht7	chitinase 7	5.37E-02	8.25E-01				
g20586	Timp	tissue inhibitor of metalloproteases	5.44E-02	9.66E-01				
g19144	mmy	mummy	5.90E-02	9.66E-01				
g19072	Exn	ephexin	6.51E-02	8.88E-01				

g21816	ple	pale	6.70E-02	7.68E-01	
g11358	CG30069	CG30069	9.82E-02	6.16E-01	
g5380	Cpr49Aa	cuticular protein 49Aa	1.26E-01	8.74E-01	○
g5356	l(3)mbn	lethal (3) malignant blood neoplasm	1.31E-01	9.66E-01	
g5320	Cpr66D	cuticular protein 66D	1.51E-01	9.66E-01	○
g14444	Cpr92F	cuticular protein 92F	1.89E-01	9.66E-01	
g1203	snsl	snustorr snarlik	1.89E-01	9.66E-01	
